# Catastrophic Antiphospholipid Syndrome Associated With Systemic Lupus Erythematosus Flare With Multiorgan Failure: A Chronicle of Cardiogenic Shock, Renal Failure, Vasculitis-Like Lesions, and Disseminated Intravascular Coagulation

**DOI:** 10.7759/cureus.17561

**Published:** 2021-08-30

**Authors:** Syed Hamza Bin Waqar, Aiman Rehan

**Affiliations:** 1 Internal Medicine, State University of New York (SUNY) Downstate Medical Center, New York, USA; 2 Internal Medicine, Dow University of Health Sciences, Karachi, PAK

**Keywords:** catastrophic antiphospholipid syndrome, systemic lupus erythematosus, vasculitis, cardiogenic shock, disseminated intravascular coagulation, dic, caps, sle

## Abstract

Catastrophic antiphospholipid syndrome (CAPS) is a rare disorder characterized by a storm of thrombosis leading to rapidly progressive multiple organ damage and thus needs to be picked earlier in the course of the disease. A higher index of suspicion is therefore mandated to initiate triple therapy to save end-organ damage. Antiphospholipid syndrome (APS) is a known association of systemic lupus erythematosus (SLE) and, when present with lupus, has the worst outcome and mainly afflicts younger cohorts. We report the case of a 33-year-old male with an extensive medical history, most notable of lupus with positive antiphospholipid antibodies complicated by nephropathy, and myocarditis presents with cardiogenic shock and progressive renal failure. The course was complicated by diffuse intra-abdominal thrombosis involving bowel, spleen, and kidneys; skin discoloration; and later disseminated intravascular coagulation (DIC). Triple therapy was initiated, which resolved the crisis, although the patient succumbed to late sequelae of infection and died of megacolon perforation. Here, we discuss the association of CAPS with SLE and a plethora of presentations, which involved but were not limited to cardiogenic shock, worsening nephropathy, mimicked vasculitis, digital cyanosis, and DIC.

## Introduction

Catastrophic antiphospholipid syndrome (CAPS), also known as Asherson syndrome, is an infrequent yet devastating complication characterized by widespread vascular occlusions [1]. It can cause a myriad of micro and macrovascular complications in a short period and displays a poor prognosis with a very high mortality rate, which is why it was deemed as being "catastrophic" [2]. Diagnosis criteria for CAPS include evidence of involvement of at least three organ systems, development of clinical manifestations simultaneously or within less than a week, histological confirmation of small-vessel occlusion, and laboratory confirmation of the presence of antiphospholipid antibodies [1]. We present our case of a 33-year-old male who succumbed to CAPS with the unique association of cardiogenic shock, vasculitis-like lesions in intra-abdominal thrombosis, digital cyanosis, and disseminated intravascular coagulation (DIC) causing multiple hindrances in management.

## Case presentation

A 33-year-old male with a significant past medical history of the congenital human immunodeficiency virus (HIV) infection, systemic lupus erythematosus (SLE) with positive anti-phospholipid antibodies (on immunosuppressive regimen), and chronic kidney disease (stage IIIb) presented to the emergency department with abdominal pain and shortness of breath. Of note, our patient had a complicated course of lupus with endomyocardial biopsy-proven lymphocytic myocarditis with residual dilated cardiomyopathy (ejection fraction of 35% and baseline brain natriuretic peptide of less than 40 pg/ml), right atrial thrombus (treated with warfarin), lupus nephritis, history of cardiac arrest in the setting of respiratory failure from unknown cause with nonspecific bronchoalveolar lavage, and multiple intubations.

**Figure 1:**
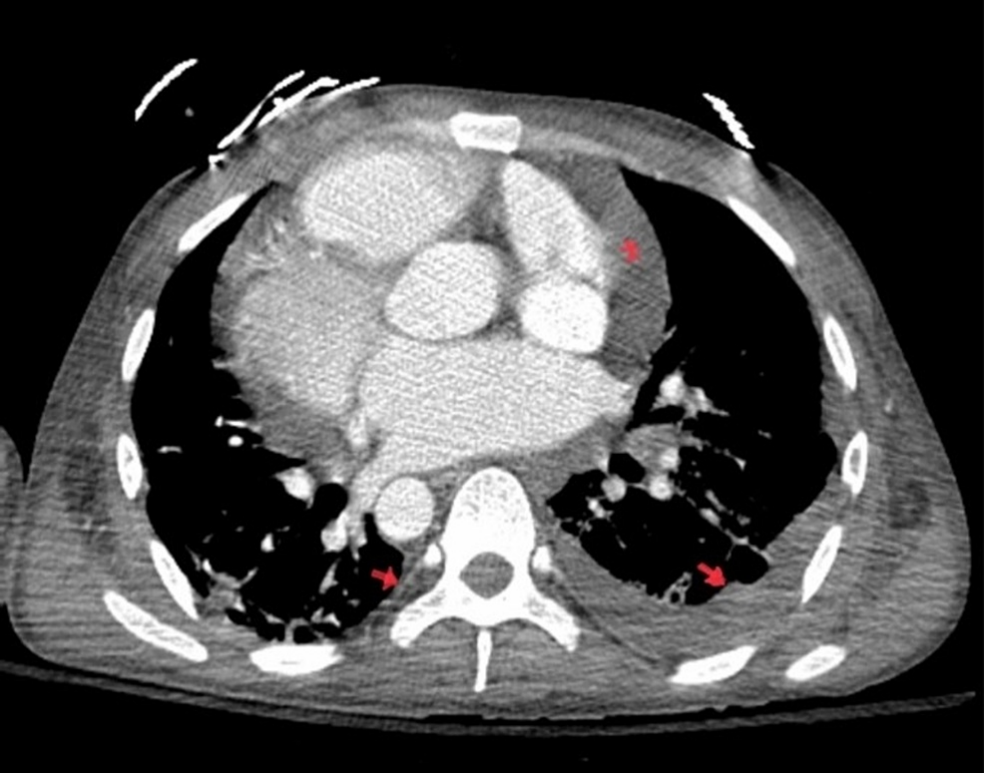
Coronal view of computed tomography (CT) chest showing moderate pericardial effusion (*) and bilateral pleural effusion with scattered basal ground-glass opacities (arrows)

**Figure 2:**
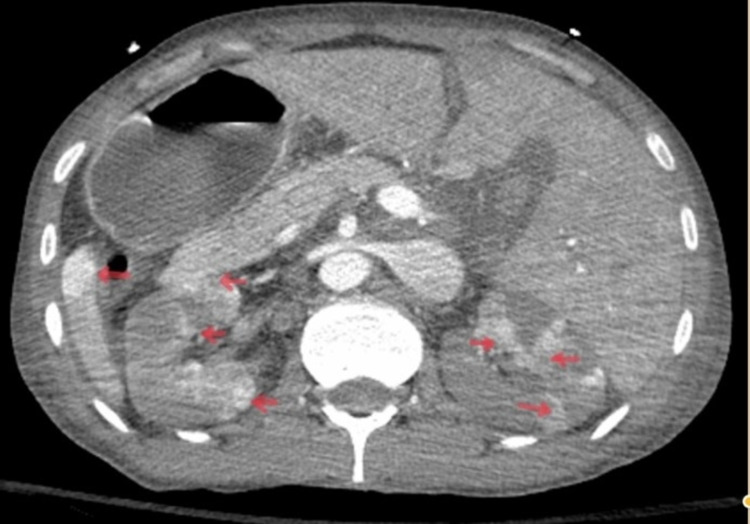
Computed tomography (CT) of abdomen/pelvis with contrast showing diffuse bilateral renal and splenic infarcts (arrows)

**Figure 3:**
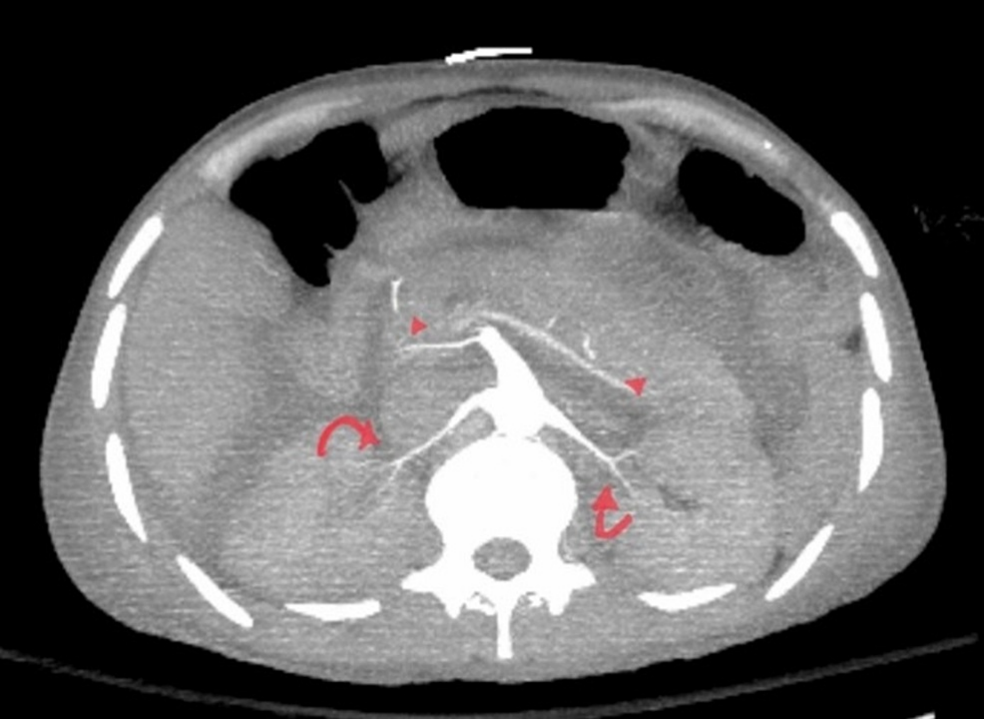
Computed tomography of abdomen/pelvis with angiogram showing tapering of celiac terminals (arrowheads) with significant narrowing of bilateral renal arteries (curled arrows)

At the presentation (first day), his saturation was in the lower 70s. Vitals were hypotensive to 70/40 mm Hg and tachycardiac to 110 bpm with tachypnea, which improved on fluids, norepinephrine, and initial course of bilevel positive airway pressure (BiPAP) ventilation with emergent intubation. Bed-side ultrasound at presentation showed a cardiogenic shock picture with volume overload. Labs at presentation showed lactic acidosis with severe transaminitis. Computed tomography (CT) of chest, abdomen, and pelvis was emergently performed, which showed pleuropericardial effusion with ground-glass opacities, tapering of pulmonary arteries without evidence of embolism, diffuse ascites with thickening of the bowel wall with adrenal hyper-enhancement along with bilateral renal and splenic infarcts with angiogram showing significant narrowing of the superior mesenteric artery (SMA), the diminutive size of inferior mesenteric artery, tapering of bilateral renal arteries, and celiac trunk branches (Figures 1-3).

The patient was transferred to the surgical intensive care unit where he underwent urgent exploratory laparotomy, which showed a viable bowel wall. Admission was further complicated with worsening renal failure with anuria (third day), requiring continuous venovenous hemodiafiltration, and presumed because of septic or ischemic acute tubular necrosis versus vascular thrombosis. We continued vancomycin and cefepime for presumed sepsis; however, culture (blood, urine, and sputum) results came out negative with slightly raised procalcitonin of 3.41 ng/mL, which made infection a less likely etiology, inconsistent with full-blown organ damage. Thus, antibiotics were discontinued within 48 hours. The focus was shifted toward lupus flare with multiorgan failure sequelae. Pulse steroids were started as the patient remained hemodynamically stable and extubated; however, thrombocytopenia worsened (fifth day), and the patient developed pain and cyanosis in the hands of bilateral upper extremities with the absent pulsation of radial arteries and feeble dorsalis pedis pulse. Concerning thrombosis and acute limb ischemia, computed tomography angiogram (CTA) of the upper and lower extremity was obtained with vascular surgery consult. Meanwhile, the patient was continued on Argatroban infusion with activated partial thromboplastin time (aPTT) titration two times above the initial baseline for presumed heparin-induced thrombocytopenia (HIT). Given normal CTA upper and lower extremity and venous and arterial duplex, no vascular intervention was made. Labs are summarized here with a detailed review of rheumatological and hematological parameters obtained (Tables 1, 2).

**Table 1:** Timeline of various laboratory tests during the course of admission ED, Emergency department; AST, aspartate transaminase; ALT, alanine aminotransferase; LDH, lactate dehydrogenase; aPTT, activated partial thromboplastin time; PT, prothrombin time; INR, international normalized ratio; BUN, blood urea nitrogen; CAPS, catastrophic antiphospholipid syndrome; DIC, disseminated intravascular coagulation; CK, creatine kinase.

Hematological and Rheumatological Parameters During Admission						
Day 1: Presentation to ED						
Parameters	Lab Value	References				
RBC	3.3	4.20-6.10 M/µL				
Hemoglobin	8.9	14.0-18.0 g/dL				
WBC	9.06	4.50-10.90 K/µL				
Neutrophil %	80.5	38.7-60.3%				
Lymphocyte %	7.9	22.4-49.0%				
Platelet’s count	231	130-400 K/µL				
AST (SGOT)	2634	10-50 U/L				
ALT (SGPT)	1528	0-41 U/L				
Lactate (arterial)	5.7	0.4-0.8 mmol/L				
Reticulocyte %	0.70%	0.5-2.9%				
HIV RNA	Undetectable	<20.0 copies/mL				
CD4 absolute count	66	489-1457/µL				
Troponin	0.125	<0.01 ng/mL				
aPTT	28	25-37 seconds				
PT	23.2	9.4-12.5 seconds				
INR	2	0.8-1.2 ratio				
BUN	62	6.0-20.0 mg/dL				
Creatinine	6.37	0.70-1.20 mg/dL				
BNP	4000	<101 pg/ml				
Day 3: Status-post laparotomy with worsening renal function						
BUN	97	6.0-20.0 mg/dL				
Creatinine	12.5	0.70-1.20 mg/dL				
WBC	8.14	4.50-10.90 K/µL				
Platelet’s count	143	130-400 K/µL				
AST (SGOT)	1048	10-50 U/L				
ALT (SGPT)	700	0-41 U/L				
Procalcitonin	3.41	0.00-0.50 ng/mL				
Days 5-14: Worsening thrombocytopenia and emerging evidence of CAPS with accelerating DIC						
Parameters	Day 5	Day 7	Day 9	Day 11	Day 14	References
RBC	2.77	2.97	2.32	2.8	2.57	4.20-6.10 M/µL
Hemoglobin	10	11.6	8.2	7.8	8	14.0-18.0 g/dL
Platelet’s count	103	96	26	75	154	130-400 K/µL
AST (SGOT)	427	242	2271	155	72	10-50 U/L
ALT (SGPT)	956	839	2023	323	186	0-41 U/L
LDH	>1000	1637	704	652	267	135-225 U/L
D-dimer	3584	4977	13499	12487	2766	0-243 ng/mL
Fibrinogen	221	152	117	146	231	200-393 mg/dL
aPTT	58	98	89	>120	40	25-37 seconds
PT	27.4	32.1	19.4	15	13.5	9.4-12.5 seconds
Anti-ds DNA	>1000		>1000		400	<29 IU/mL
C3 levels	56		54	63	74	86-184 mg/dL
C4 levels	8		5		8	20-58 md/dL
CK level	70		228		65	20-200 ng/mL

**Table 2:** Extensive hematological and rheumatological profile of our patient with the diagnosis of probable catastrophic antiphospholipid syndrome with lupus

Extensive Hematological and Rheumatological Profile Testing During Admission		
Parameters	Patient’s Lab	References
C-reactive protein	53.41	1.0-4.0 mg/L
Tumor necrosis factor alpha (TNF-alpha)	7.2	<1.7 pg/mL
Interleukin-2 (IL-2)	<2.1	<2.1 pg/mL
IL-2 receptor CD25 soluble	1686.0	175.3-858.2 pg/mL
Interleukin 10 (IL-10)	8.4	<2.8 pg/mL
Antinuclear antibody (ANA) pattern	Homogenous	Undetectable
ANA titers	>1:1280	<1:20: Negative
Extractable nuclear antigen (ENA) Smith antibody	0.2	<0.9 AI
ENA-(nuclear riboprotein) ribonucleoprotein (RNP) antibody	0.9	<0.9 AI
Anticentromere B antibody	<0.2	0.0-0.9 AI
Anti-Scl70 antibody	<0.2	0.0-0.9 AI
Anti-Jo1 antibody	<0.2	0.0-0.9 AI
Rheumatoid factor antibody	<15	0-15 U
Anticyclic citrullinated peptide (CCP) antibody	7	0-19 U
Antimyeloperoxidase (MPO) antibody	<9.0	0.0-9.0 U/mL
Antiproteinase 3 antibody	<3.5	0.0-3.5 U/mL
Cytoplasmic, antineutrophil cytoplasmic antibodies (cANCA)	<1:20	<1:20: Negative
Perinuclear, antineutrophil cytoplasmic antibodies (pANCA)	<1:20	<1:20: Negative
Angiotensinogen converting enzyme (ACE) levels	54	14-82 U/L
Lupus anticoagulant	Moderately positive (reported thrice)	Undetectable
Beta 2 Glycoprotein 1 IgG antibody	<5.0	<20.0 SGU
Cardiolipin antibody IgA	<9	0.0-12.5 APL U/mL
Cardiolipin antibody IgG	16.8 (reported thrice)	0.0-12.5 GPL U/mL
Cardiolipin antibody IgM	84.2 (reported thrice)	0.0-12.5 MPL U/mL (>80 MPL: strongly positive)
HIT IgG assay	Undetectable	Undetectable
ADAMTS13 activity	69.6	>66.8%

Finally, a multidisciplinary round was made, and final diagnosis of the probable CAPS was put forward as the patient developed more than three organ system involvement (kidney, heart, bowel, skin, spleen, and lungs) within a week of presentation despite delays in diagnosis because of the complex presentation along with positive antiphospholipid antibodies. Consent could not be obtained for a small-vessel biopsy. The patient was thus started on plasma exchange (seventh day) for a total of five days (with the removal of one to one-and-half liter volume) with 100% supplementation of fresh frozen plasma with the continuation of prednisone after pulse steroid. Argatroban infusion was also stopped as the HIT panel came back negative and transitioned to heparin drip. However, after a day of plasma exchange, the patient developed bleeding from his intravenous lines with worsening mental status and respiratory compromise mandating another intubation. Platelet count dropped to less than 30,000/μL, and the peripheral smear obtained showed heavily granulated neutrophils with progressive erythrocytic crowding, few schistocytes per high power field, nucleated red blood cells, and significantly decreased platelets suggestive of early DIC that was accelerating in the context of SLE with probable CAPS. CT scan of the head and magnetic resonance angiograms (MRA) of head and neck were non-contributory and did not show any ischemia, thrombosis, or hemorrhage. Supportive management was done with platelet transfusion with the goal of above 100,000 and cryoprecipitate, with the suspension of heparin drip and initiation of a prophylactic dose of heparin. Plasma exchange was continued, and after recovery of platelets, he was transitioned back to heparin drip and later warfarin after 48 hours (tolerated without bleeding), extubated, and started back on his immunosuppressive regimen, which was previously held. The patient showed improvement clinically on hematological parameters and was later downgraded to a non-critical set-up. However, during the same admission, the patient developed diarrhea because of *Clostridium difficile* and later developed megacolon, for which surgery was recommended, but the patient went against it. The course was complicated with perforation, which led to his death.

## Discussion

CAPS is a thrombotic storm causing a myriad of micro and macrovascular complications in a short span leading to multiorgan failure and thus associated with a very high mortality rate [1,2].

Triggers for CAPS have been recognized, with most triggers likely being an underlying infection (48%), with SLE contributing to only less than 3% of the causation [3,4]. As per the registry, SLE-associated APS is more common in younger patients and is associated with a higher mortality rate than primary antiphospholipid syndrome (APS) (48% versus 33%) [4].

No matter what the trigger, it is essential to recognize the hallmarks of CAPS as early detection can be lifesaving. As per the international registry for CAPS, intra-abdominal thrombotic complications herald as abdominal pain or discomfort. In the SLE cohort, kidney (74%), CNS (67%), lung (65%), heart (56%), skin (55%), liver (39%), and GI (21%) were the most involved organ systems. Of note, hematological parameters can also give clues to diagnosis with thrombocytopenia (74%), anti-cardiolipin (aCL), IgG (83%), and anti-beta-2-glycoprotein 1 (aβ2GPI) IgG (92%) among the most frequent anomalies [4].

To understand the therapeutics for CAPS, it is crucial to recognize the double-hit model of pathogenesis. The first hit is sought to be a genetic susceptibility of thrombophilia (antiphospholipid antibodies), with the second hit being the triggers (be it a flare or infection) [5]. To date, we do not have sufficient data to guide treatment for CAPS from controlled trials; however, triple therapy involving anticoagulation (first hit), glucocorticoids, and plasmapheresis (second hit) have been supported in various studies [6,7]. Rituximab has been used in refractory cases of CAPS [8]. Of note, intravenous cyclophosphamide has been noted to be of benefit in SLE-associated CAPS [7]. Eculizumab has also been used in relapses and has been shown to maintain sustained remissions in refractory primary CAPS [9].

In our case, the patient presented with cardiogenic shock, which progressed to renal failure. Trigger remained unchecked as CAPS diagnosis remained a conundrum despite various hints and later manifested as diffuse intra-abdominal thrombosis in its storm driving to its diagnosis, although unfortunately ending up in DIC leading to another complication.

## Conclusions

This case highlights the importance of having a higher suspicion of CAPS in patients with SLE, especially those with positive antiphospholipid antibodies without an apparent diagnosis of antiphospholipid syndrome. Our case reports the rarest of presentations of CAPS compounded together in a patient having multiorgan failure, which made it extremely complex to decide on its diagnosis. Close liaison between hematologist, rheumatologist, and critical care is necessary, and earlier treatment with triple therapy might prove to be lifesaving.
